# Porcine Zygote Injection with Cas9/sgRNA Results in *DMD*-Modified Pig with Muscle Dystrophy

**DOI:** 10.3390/ijms17101668

**Published:** 2016-10-09

**Authors:** Hong-Hao Yu, Heng Zhao, Yu-Bo Qing, Wei-Rong Pan, Bao-Yu Jia, Hong-Ye Zhao, Xing-Xu Huang, Hong-Jiang Wei

**Affiliations:** 1 School of Life Science and Technology, ShanghaiTech University, 100 Haike Rd., Pudong New Area, Shanghai 201210, China; geneyhh@126.com; 2 State Key Laboratory for Conservation and Utilization of Bio-Resources in Yunnan, Yunnan Agricultural University, Kunming 650201, China; hengzhao2014@126.com (H.Z.); qingyubo20@163.com (Y.-B.Q.); jiabaoyu2009@163.com (B.-Y.J.); hyzhao2000@126.com (H.-Y.Z.); 3 College of Animal Science and Technology, Yunnan Agricultural University, Kunming 650201, China; pwr2000@sina.com; 4 Research Center of Life Science, Yulin University, Yulin 719000, China

**Keywords:** CRISPR/Cas9, *DMD*, pig, disease model, gene editing

## Abstract

Dystrophinopathy, including Duchenne muscle dystrophy (DMD) and Becker muscle dystrophy (BMD) is an incurable X-linked hereditary muscle dystrophy caused by a mutation in the *DMD* gene in coding dystrophin. Advances in further understanding DMD/BMD for therapy are expected. Studies on mdx mice and dogs with muscle dystrophy provide limited insight into DMD disease mechanisms and therapeutic testing because of the different pathological manifestations. Miniature pigs share similar physiology and anatomy with humans and are thus an excellent animal model of human disease. Here, we successfully achieved precise *DMD* targeting in Chinese Diannan miniature pigs by co-injecting zygotes with Cas9 mRNA and sgRNA targeting *DMD*. Two piglets were obtained after embryo transfer, one of piglets was identified as *DMD*-modified individual via traditional cloning, sequencing and T7EN1 cleavage assay. An examination of targeting rates in the *DMD*-modified piglet revealed that sgRNA:Cas9-mediated on-target mosaic mutations were 70% and 60% of dystrophin alleles in skeletal and smooth muscle, respectively. Meanwhile, no detectable off-target mutations were found, highlighting the high specificity of genetic modification using CRISPR/Cas9. The *DMD*-modified piglet exhibited degenerative and disordered phenotypes in skeletal and cardiac muscle, and declining thickness of smooth muscle in the stomach and intestine. In conclusion, we successfully generated myopathy animal model by modifying the *DMD* via CRISPR/Cas9 system in a miniature pig.

## 1. Introduction

The *DMD* mutation is the genetic factor responsible for the development of Duchenne muscular dystrophy (DMD), an X-linked hereditary muscle dystrophy [1,2]. Absence of dystrophin coded by *DMD* compromises the stability of the sarcolemma surrounding muscle fibers, leading to rupture of the muscle cell membrane and leakiness that induces necrosis in myofibrils, subsequent progressive tissue fibrosis, replacement by fat and loss of functional capacity [3]. DMD affects 1 in 3500–5000 boys [4,5]. The clinical course of DMD is severe and progressive, starting with muscle weakness at 5 years of age and loss of ambulation ability at about 12 years; death occurs due to respiratory failure or cardiomyopathy in the late teens [1,6]. No effective therapeutic treatment is available for patients suffering from DMD. Thus, a detailed understanding of DMD is necessary for developing effective therapies.

Several animal models manifesting the phenotype observed in DMD disease have been generated in the laboratory or identified in nature, including mdx mice and dogs with X-linked muscular dystrophy (cxmd) [7]. These models generally show the pathological alterations observed in human patients and have been used to understand the pathological mechanism of DMD and to test candidate therapies [8,9]. The advantages and disadvantages of using mdx mice and dogs with cxmd are obvious [10,11]. Although mdx mice are easy to maintain and breed, the skeletal muscle degenerative phenotypes are much milder than those of DMD in humans. Dogs with cxmd reflect the pathological severity of human DMD, with early onset muscle weakness, lethal respiratory distress, and cardiomyopathy. Unfortunately, the phenotypes can vary among dogs with cxmd [12]. *DMD*-modified monkeys and rats have been generated using the CRISPR/Cas9 gene editing tool to provide alternative models for investigating treatments, and understanding the pathological mechanism of DMD. The monkey is an excellent DMD model. However, experimental monkeys are limited, and considerable labor is required to maintain and breed them; these limit the wide application of the DMD monkey model. Rats have historically been considered as a useful species for investigating new medicines to treat human diseases, particularly with respect to evaluating pharmacological effects and toxicity [13]. The *DMD* null mutation rat also exhibits degenerative/regenerative phenotypes in skeletal muscle, heart [12]. However, due to the distant phylogenetic relationship, and different physiological and anatomical features between rats and humans, the pathology recapitulating the clinical traits of patients with DMD should be further investigated in *DMD* null mutation rats.

To develop novel therapeutic strategies, animal models that accurately recapitulate DMD are necessary. Pigs are an ideal animal model for human disease because their physiological and anatomical characteristics are more similar to humans than are those of mice, rats and dogs. These similarities increase the likelihood of a more accurate recapitulation of the DMD. Meanwhile, genetically modified pigs have great promise in biomedical research [14,15]. The CRISPR/Cas9 system has been demonstrated to be a highly efficient genomic targeting tool for generating gene-modified animal models [12,16,17,18,19,20]. Our previous success in generating a genetically modified pig using the CRISPR/Cas9 system inspired us to generate a DMD pig model [17,21]. Here, we employed the CRISPR/Cas9 system to knockout *DMD* to determine whether pigs lacking dystrophin could function as an animal model by recapitulating the human DMD phenotypes.

## 2. Results

### 2.1. Validation of sgRNA Targeting DMD

The Diannan miniature pig is well known as an exclusive native breed in Yunnan Province, China. These pigs are famous for their early sexual maturation and suitable full-grown body weight, which makes this strain ideal for generating a human disease model [22,23].

**Figure 1:**
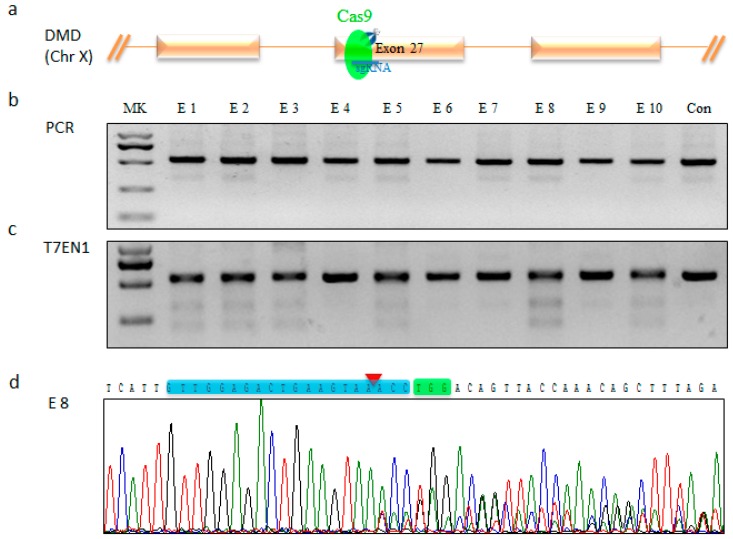
Evaluation of *DMD* sgRNA:Cas9-mediated modifications of *DMD* in pig parthenogenetic embryos. (**a**) Schematic diagram of the pig *DMD* partial protein coding region and the *DMD* sgRNA:Cas9 targeting site; (**b**) Polymerase chain reaction (PCR) products of the *DMD* targeting region in pig embryos. MK, DNA marker; Con, PCR product of the targeted region amplified from wild-type pig; (**c**) Detection of sgRNA:Cas9-mediated on-target cleavage of *DMD* by T7EN1 cleavage assay in pig embryos; (**d**) Representative sequencing chromatographs of PCR product. Blue background indicates the *DMD* sgRNA sequence. Red triangle indicates cleavage site mediated by *DMD* sgRNA:Cas9. Green background indicates PAM construct.

To introduce mutations in *DMD* gene located in the X chromosome, which consists of a total of 79 exons in Diannan miniature pig, one sgRNA targeting *DMD* exon 27 (Figure 1a) was designed as described previously [24]. In order to investigate the targeting effect of the designed sgRNA in embryos, Cas9 mRNA and *DMD* sgRNA were transcribed *in vitro* using T7 RNA polymerase [25]. Cas9 mRNA (20 ng/µL) and sgRNA (10 ng/µL) were pooled and micro-injected into 455 pig parthenogenetic embryos (Table S1). Sixty embryos developed normally to the blastocyst stage with similar developmental rates compared with that of H_2_O injection groups, but lower than that of the untreated group, Cas9/sgRNA injection group: 20% (60/300) vs. H_2_O injection group: 17.8% (29/163) vs. untreated group: 35.2% (55/156) (Table S1). The number of cells comprising the parthenogenetic blastocyst embryos further revealed excellent developmental ability (Cas9/sgRNA injection group: 55.5 vs. H_2_O injection group: 58.7 vs. untreated group: 57.5, Table S1, Figure S1). Subsequently, pig genomic DNA was isolated from 10 blastocyst stage embryos harvested 168 h after micro-injection, and the region around the target site was amplified by polymerase chain reaction (PCR) (Figure 1b). The above PCR products were digested by T7EN1 enzyme after denaturation and anneal. T7EN1 cleavage assay showed that 6 of 10 samples (60%) displayed cleavage bands (Figure 1c). Sequencing of PCR products showed overlapping peaks on chromatographs (Figure 1d), suggesting different genotypes occurred at the target site. These data demonstrate that the designed sgRNA worked well with Cas9 for targeting *DMD* in pig embryos.

### 2.2. Generation of DMD-Modified Pigs by Injecting Zygotes with Cas9/sgRNA

**Figure 2:**
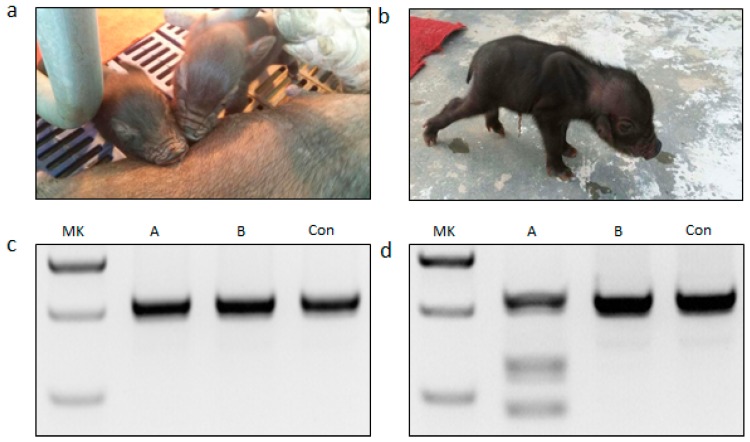
Detection of *DMD* sgRNA:Cas9-mediated modifications of *DMD* in funicle tissues of founder pigs. (**a**) Photograph of newborn founders; (**b**) *DMD*-modified founder A; (**c**) PCR products of the *DMD* targeted region amplified from founder pigs. MK, DNA marker; A, founder A; B, founder B; Con, PCR product of targeted region amplified from wild-type pig; (**d**) Detection of sgRNA:Cas9-mediated on-target cleavage of *DMD* by the T7EN1 cleavage assay in founder pigs. Con, PCR product of targeted region amplified from wild-type pig, digested by T7EN1.

After verifying the targeting effect of the designed sgRNA and Cas9 mRNA in pig parthenogenetic embryos, we next generated *DMD*-modified Diannan miniature pigs. A total of 98 1-cell stage embryos were surgically collected from 19 mated embryo donor pigs. Cas9 mRNA (20 ng/µL) and sgRNA (10 ng/µL) mixtures were injected into the cytoplasm of embryonic cells. After injection, ninety-eight embryos were immediately transferred into eight surrogate females. After 114 days full-term pregnancy, one surrogate female (12.5%; 1/8) successfully delivered two offspring called founder A and founder B (Figure 2a,b, Table S2). The funicle tissues of the two piglets were collected for identification of *DMD*-modification. Unfortunately, founder A died 52 days after birth and founder B died 3 days after birth, and we collected skeletal muscle tissues, smooth muscles, fat, brain and skin etc. for further use.

### 2.3. Genotype Analysis of DMD-Modified Pigs

**Figure 3:**
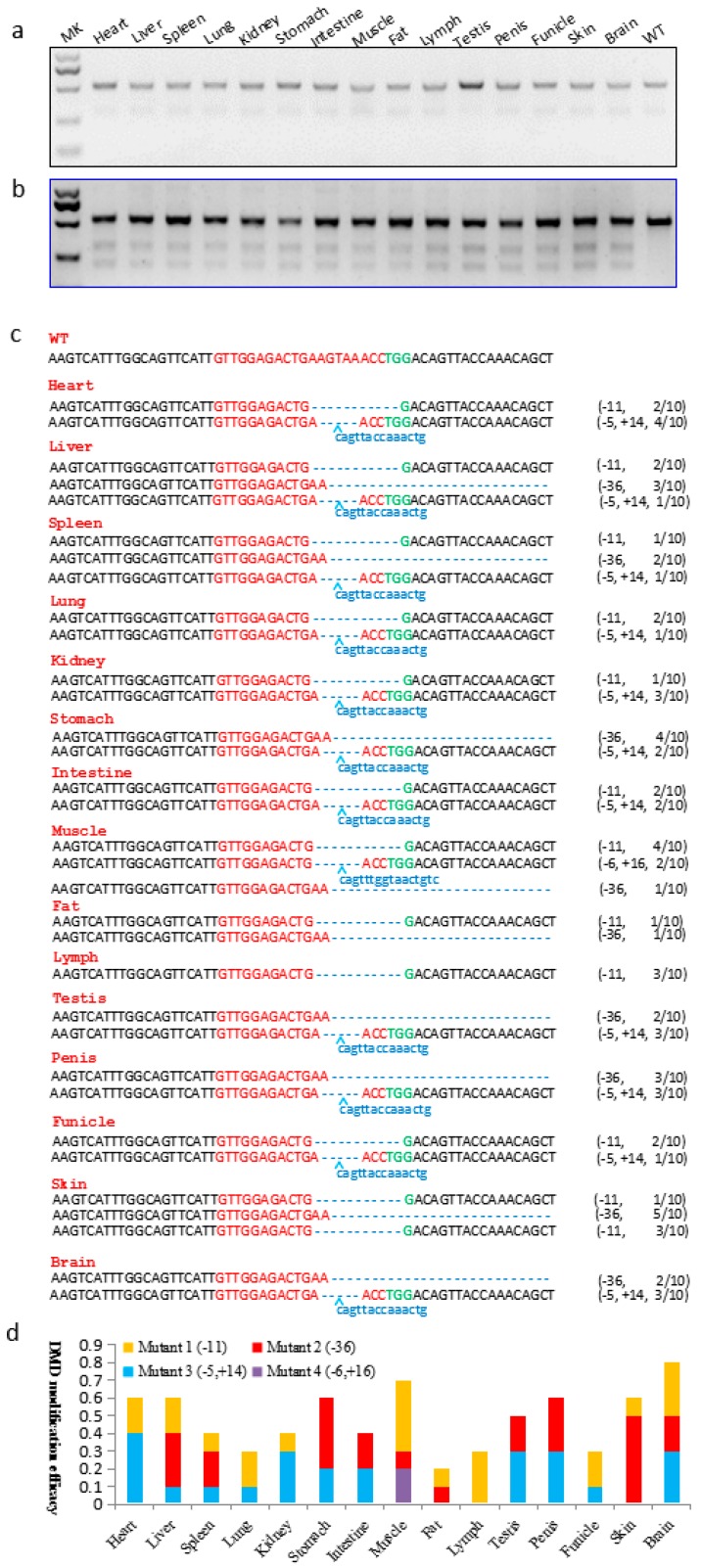
sgRNA:Cas9-mediated *DMD* modifications in different tissues of founder A. (**a**) PCR products of the *DMD* targeting region. WT, PCR product amplified from wild-type pig; (**b**) Detection of sgRNA:Cas9-mediated on-target cleavage of *DMD* by the T7EN1 cleavage assay; (**c**) Modified *DMD* sequences detected in founder A. WT, wild-type DNA sequence. Sequences complementary to sgRNA targeting site are labeled in red, and PAM sequences are in green; the mutations in blue, lower case indicates the inserted base, deletions (−), insertions (+); (**d**) sgRNA:Cas9-mediated *DMD* modification frequency in different founder A tissues. Mutant 1, 11 base pair deletion; Mutant 2, 36 base pair deletion; Mutant 3, five base pair deletion and 14 base pair insertion; Mutant 4, 6 base pair deletion and 16 base pair insertion.

The genomic DNA of the funicle tissues from two piglets were extracted for genotyping. The region around the target site was amplified by PCR (Figure 2c). The PCR product amplified from founder A funicle (Figure 2d) digested by T7 endonuclease I (T7EN1) showed expected cleavage bands, but not founder B (Figure 2d), suggesting that *DMD* modifications had occurred in founder A. Sanger sequencing of PCR production from founder B also revealed the targeting site in *DMD* was not disrupted by CRISPR/Cas9 system. Mosaic mutations in the gene modified mammals induced by injection of Cas9 mRNA and sgRNA at one-cell stage embryo commonly occurred, which resulted from the prolonged effects of the CRISPR/Cas9 system after one cell cleavage. Thus, it is important to further investigate Cas9:sgRNA-mediated *DMD* modifications distributed in porcine tissues, such as muscle. A high rate would indicate that most of the *DMD* gene has been disrupted to yield the loss of function of dystrophin. The T7EN1 cleavage assay and TA-cloning sequencing were conducted to analyze the genotypes in 15 tissues from founder A, including heart, liver, spleen, lung, kidney, stomach, intestine, muscle, fat, lymph, testis, penis, funicle, skin, and brain (Figure 3a,b). The expected additional bands were all observed in 15 tissues from founder A after T7EN1 cleavage assay, indicating that the CRISPR/Cas9 system can mediate efficient *DMD* editing in detected tissues. As expected, founder A was a mosaic *DMD*-modified individual, and the *DMD* modification efficiency was 20%–80% in the 15 tissues (Figure 3d and Table S3). The *DMD* modification efficiency was 70% and 40% in skeletal and smooth muscle, respectively (Figure 3d and Table S3). TA-cloning sequencing showed four types of indels (mutant 1: deletion of 11 bp; mutant 2: deletion of 36 bp; mutant 3: deletion of 5 bp and insertion of 14 bp; mutant 4: deletion 6 bp and insertion 16 bp) found at the *DMD* target site (Figure 3c), and the frequency of three types of indels mediated by CRISPR/Cas9 system varied in different tissues of founder A (Figure 3d and Table S3). Mutant 1 and mutant 4 caused frame shift of the coding sequence of the *DMD* gene. The efficiency of the *DMD* null mutation was 60% in skeletal muscle.

### 2.4. Off-Target Detection

Off-target effect of CRISPR/Cas9 system has remained a challenge since establishment of this technology. Thus, we determined whether an off-target effect occurred in the *DMD*-modified pig. We identified the 14 most likely off-target sites (OT 1–14) (Table S4) in other regions of founder A genome that differed by five or fewer nucleotides in the *DMD* target site and amplified these DNA fragments via PCR from 14 tissues types of founder A. The PCR products amplified from OT sites 1–14 were subjected to the T7EN1 cleavage assay (Figure S2) and DNA sequencing analysis. No cleavage bands were found at any potential OT sites. In the sequencing results no overlap peak was found and no DNA mutation occurred at OT sites, indicating that potential off-target mutations were not induced by CRISPR/Cas9 system.

### 2.5. Phenotypic Analysis of the DMD-Modified Pig

**Figure 4:**
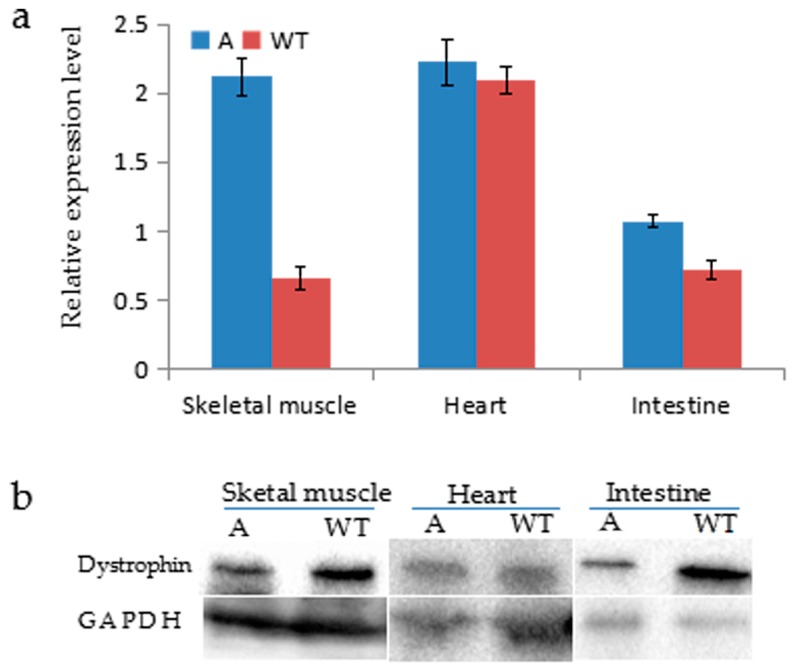

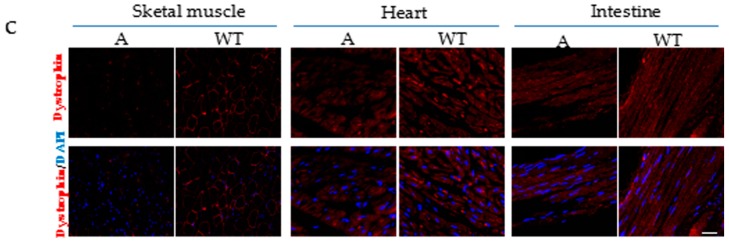
Expression of dystrophin in muscle tissues. (**a**) The relative expression of *DMD* mRNA in founder A and the age-matched wild-type pigs; (**b**) Western blotting for Dystrophin and GAPDH in biceps femoris muscle, heart and intestine of *DMD*-modified founder A and WT; (**c**) Immunostaining for dystrophin in biceps femoris muscle, heart and intestinal of *DMD*-modified founder A and WT. A indicates founder A, WT indicates wild-type pig of the same age. Frozen section. Scale bar: 50 µm.

**Figure 5:**
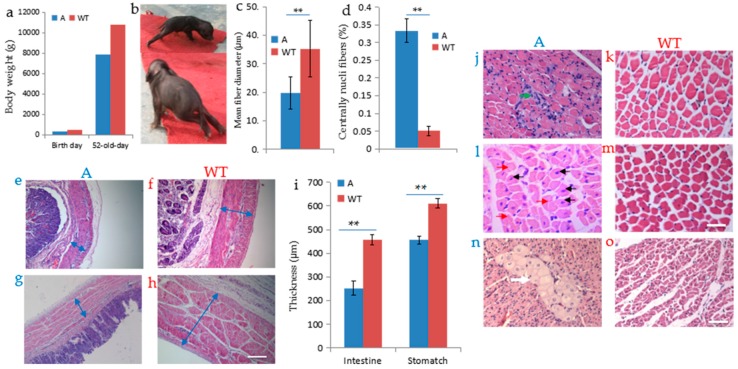
*DMD* deficiency resulted in pathological alterations in the *DMD*-modified pig. (**a**) The birth weight (480 g) and body weight at 52 days (7809 g) of founder A were lower than that of the wild-type piglet (birth weight 575 g and body weight at 52 days was 110,200 g); (**b**) Behavioral performance of muscle weakness of founder A; (**c**) Mean minimal Feret’s diameter of muscle fibers in biceps femoris muscle of 52-day-old founder A and WT; (**d**) Proportion of muscle fibers with central nuclei in deltoid muscle of 52-day-old founder A and WT. ** *p* < 0.01; (**e**–**h**,**j**–**o**) Haematoxylin and eosin (H&E) staining, paraffin section; (**e**–**i**) Smooth muscle thickness of stomach (**e**) and intestine (**g**) were dramatically decreased compared with that of WT (**f**,**h**). Blue arrows indicate the thickness of stomach and intestine; (**j**) Cross-section of biceps femoris muscle revealed disordered myofiber construction and the green arrow indicated the aggregated nuclei; (**l**) Black arrows indicated the rounded myofibers with central nuclei, and red arrows indicated the necrosis of myofibers in deltoid muscle; (**n**) Multifocal area of pale discoloration in cardiac muscle was observed in section; (**k**,**m**,**o**) histological section of skeletal muscle and cardiac muscle of wild-type. Scale bars: 50 µm (**e**–**h**,**j**–**m**),100 µm (**n**,**o**).

Given the nature of mosaic mutations created by CRISPR/Cas9 system, it is important to determine whether the *DMD* mosaic mutations will result in dystrophin dysfunction and induce the DMD phenotypes characterized by muscle degeneration due to lack of dystrophin [2,26,27]. We first investigated skeletal and smooth muscle dystrophin expression in the targeted pig and compared it with that in muscle from wild-type pig of the same age (52 days old). Although the *DMD* mRNA relative expression levels were higher than control in biceps femoris muscle, heart and intestine (Figure 4a), western blotting and immunostaining with antibody for dystrophin indicated the dystrophin in founder A was relatively low compared to that of wild type (Figure 4b,c). Next, we investigated the pathologies of the *DMD*-modified pig. Founder A displayed lower body weight compared with that of wild-type pig (Figure 5a), and muscle weakness reflecting in movement (Figure 5b). We observed the difference in the mean minimal Feret’s diameter of myofiber size in skeletal muscle compared with that of control (Figure 5c). A greater proportion of muscle fibers with central nuclei was found in skeletal muscle of the *DMD*-modified pig (Figure 5d,l). The thickness of the smooth muscle layer of the stomach and intestine decreased drastically compared with that of the wild-type pig (Figure 5e–i). Hematoxylin and eosin (H&E) staining also revealed extensive disruption of muscle structure, including disordered myofibers (Figure 5j) in skeletal muscle tissues. In addition, the muscle cell nuclei often aggregated together due to necrosis of the muscle fibers (Figure 5j). Multifocal areas of pale discoloration in cardiac muscle in founder A were found (Figure 5n). In summary, the pathological analysis suggested that the *DMD*-modified pig displayed characteristic phenotypes of muscular dystrophy due to the deficiency of dystrophin.

## 3. Discussion

Injecting Cas9 mRNA and sgRNA into zygotes has proven to be an efficient technological method for generating gene-modified mammals, including pigs, goats, and monkeys [16,17,18]. Here, we designed sgRNA targeting *DMD* and detected a targeting efficacy of 60% in pig parthenogenetic embryos. Further investigation of embryonic development revealed that Cas9 mRNA and sgRNA are nontoxic to embryos, as in other species [16,17,25]. However, physical damage induced by the injection impedes development. 

This success of sgRNA/Cas9-mediated *DMD* modification of embryos inspired us to generate *DMD*-modified pigs by microinjection, and the targeting efficacy of *DMD* was 50% in two piglets. Immunostaining and western blotting demonstrated that the quantity of dystrophin protein in the mutant pigs was relatively low compared to that of the wild-type pig. This result demonstrates that *DMD* knockout was achieved in the *DMD*-modified pig. Meanwhile, the disruption of dystrophin indicates that CRISPR/Cas9 is an efficient gene editing tool for modified gene in pig by injection of zygote.

Off-target mutagenesis is a major concern when using the CRISPR/Cas9 system, and CRISPR/Cas9-induced off-target mutations are heritable in mice and rats [28]. Therefore, we comprehensively detected the 14 potential off-target sites in 15 tissues. As expected, no off-target mutation was detected in the *DMD*-modified founder using the sgRNA, suggesting that the CRISPR/Cas9 system achieved specific gene targeting, resulting in *DMD-*specific phenotypes.

Various animal models have accelerated the advances in understanding of the path physiology of muscular dystrophy and allowed the progress of some promising approaches to therapy [29,30,31,32]. However, these animal models have species-specific differences in their clinical courses and phenotypes. Hypertrophic muscle with centralized nuclei is the common pathological feature in DMD/BMD animal disease model, which is found in DMD patients [33]. The hypertrophic muscle fibers with centralized nuclei were also found in our *DMD*-modified pig. Meanwhile, *DMD*-modified pigs show a characteristic reduction in movement ability, which is comparable with the early symptoms of DMD/BMD patients. It appears that *DMD*-modified pigs exhibit the pathological and functional hallmarks of the human disease. Furthermore, the lack of dystrophin also results in cardiac muscle defects and eventual death. Investigation of the *DMD*-modified pig found pathological changes. Taken together, the *DMD*-modified pig appears to be an appropriate model of DMD/BMD as ascertained by muscular dystrophy and the behavioral performance.

In conclusion, we successfully targeted *DMD* by injecting zygotes using the CRISPR/Cas9 system. The *DMD*-modified miniature pig is a reliable and useful animal disease model that recapitulates the DMD/BMD morphological phenotypes in humans.

## 4. Materials and Methods

### 4.1. Animals

The animals used in this study were maintained at the Laboratory Animal Center of Yunnan Agricultural University. All experiments involving animals were approved by the Institutional Animal Care and Use Committee of Yunnan Agricultural University (Permission code: YAUACUC01; Data of publication: 10 July 2013).

### 4.2. Construct and in Vitro Transcription

Two complementary DNA oligos for generating sgRNA, listed in Table S5, were annealed. Subsequently, the double strand DNA coding the sgRNA was subcloned into the pUC57-T7-gRNA vector as described [25] to construct the recombinant vector and prepare sgRNA by in vitro transcription. The completely linearized recombinant vector was digested by the *Dra*I endonuclease and used as a template. The sgRNA targeting *DMD* was generated via in vitro transcription using the MEGAshortscript kit (Ambion, Austin, TX, USA) and purified using the MEGAClear kit (Ambion) according to the manufacturer’s instructions. Cas9 mRNAs were produced and purified as described previously [25].

### 4.3. Pig Parthenogenetic Embryo Cas9/sgRNA Injection

Oocytes were collected and matured by in vitro culture, as described previously [34]. Cumulus-oocyte complexes (COCs) with at least three layers of compacted cumulus cells were selected, and approximately 50 COCs were cultured in 200 µL in vitro maturation (IVM) medium at 38.5 °C in a 5% CO_2_ atmosphere (APC-30D; ASTEC, Tokyo, Japan) and saturated humidity for 42–44 h. After IVM, COCs with expanded cumulus cells were treated briefly with 0.1% (*w*/*v*) hyaluronidase, and the cumulus cells were removed by gentle pipetting. The mature oocytes with an extruded polar body were selected and activated with a single pulse of 150 V/mm for 100 µs in an activation medium. The Cas9/sgRNA mixture was microinjected after activation for 1 h, as described previously [35]. The injected parthenogenetic embryos were cultured in porcine zygote medium-3 as described previously [34]. The cleavage rate of parthenogenetic embryos was counted 48 h post-activation, and the blastocysts were harvested 168 h post-activation. The genomic DNA was isolated from single parthenogenetic blastocysts by incubating individual embryos in lysis buffer, as described previously [35]. A primer pair set (Table S6) was used to amplify the modified *DMD* alleles in injected embryos by PCR using genomic DNA as the template, and the amplification products were subjected to T7EN1 analysis after purification with a gel extraction kit (Qiagen, Hilden, Germany).

### 4.4. Production of DMD-Modified Pigs by Injecting Zygotes with Cas9/sgRNA

Healthy pigs with normally estrus cycles were used as donors for single cell stage embryo collection. Embryo collection from mated female pigs and treatment of embryo donors were conducted as described previously [36]. Single cell stage embryos (18–24 h after the last mating) were surgically obtained and were immediately cultured in TCM199 medium (Thermo Fisher Scientific, Shanghai, China). Cas9 mRNA and sgRNA targeting *DMD* were mixed to final concentrations of 20 and 10 ng/µL. Then, the mixture was injected into the cytoplasm of single cell embryos by EppendorfFemtoJect System. The injection time, injection pressure and compensatory pressure were 0.1 s, 45 and 7 kpa, respectively. Cas9 mRNA and sgRNA were microinjected into cytoplasm of embryos in TCM199 medium on the heated platform of an Olympus micro-manipulation system ON3. The injected embryos were transferred into synchronized foster mother sows immediately after micro-injection, as described previously [36]. Two piglets were delivered naturally 113 days after embryo transfer.

### 4.5. T7EN1 Cleavage Assay and Sequencing

The different tissues collected from piglets were digested in lysis buffer (0.4 M NaCl, 2 µM EDTA, 1% SDS, 10 µM Tris-HCl, and 100 µg/mL Proteinase K) overnight. Genomic DNA of the sample was extracted from the lysate with phenol-chloroform and recovered by alcohol precipitation. The T7EN1 cleavage assay was performed as described previously [25]. PrimerSTAR HS DNA polymerase (DR010A; Takara Bio, Shiga, Japan) was used to amplify the targeted fragments around the sgRNA targeting site from the extracted genomic DNA, and the PCR products were purified with a cleanup kit (AP-PCR-50; Axygen, Union City, CA, USA). The primers for amplifying the *DMD* targeted fragments are listed in Table S6. A mixture of 50 ng wild-type purified PCR product and 150 ng of the tested purified PCR product was denatured and re-annealed in NEBuffer 2 (New England Biolabs, Ipswich, MA, USA) using a thermocycler. The PCR products were digested with T7EN1 (M0302L; New England Biolabs, Ipswich, MA, USA) for 30 min at 37 °C and separated by 2.5% agarose gel electrophoresis. The PCR products with mutations detected by the T7EN1 cleavage assay were sub-cloned into the T vector (D103A; Takara Bio). Colonies for each sample were picked up randomly and sequenced with the M13F (-47) (M13F (-47): 5′-CGCCAGGGTTTTCCCAGTCACGAC-3′) primer.

### 4.6. Off-Target Assay

Potential off-target loci were searched for using the SeqMap open tool [37]. The mismatch parameter for the off-target sequence was set to “NGG” and chosen as PAM. Sites with total mismatches <5 were chosen as potential off-target sites for testing. The predicted off-target sites are listed in Table S4. The selected potential off-target sites were PCR amplified using genomic DNA from founder A as the template. The primers for amplifying the off-target sites are listed in Table S7. Then, the PCR products were subject to the T7EN1 cleavage assay as described above.

### 4.7. Immunohistochemistry and Immunofluorescence

Tissues from founder A and the wild-type pigs were fixed in 4% paraformaldehyde (PFA) for 48 h prior to immunohistochemistry, and 15% and 30% sucrose solutions were used to dehydrate the respective tissues. Frozen 8-µm tissue sections were prepared transversely. After blocking with 5% normal goat serum in phosphate buffered saline containing 0.3% Triton X-100 (Sigma, St. Louis, MO, USA), the cryosections were incubated overnight with (1:50) anti-dystrophin antibody (ab15277; Abcam, Shanghai, China) at 4 °C for 12 h, followed by washing and incubation with secondary antibody (Alexa Fluor 594-conjugated, goat anti-rabbit IgG, Jackson immuno, 1:200) for 2 h at room temperature. Nuclei were counterstained with DAPI. Photographs were taken under a fluorescence microscope (FV1000; Olympus, Tokyo, Japan).

For western blotting, biceps femoris muscle, cardiac muscle and intestine tissues of the 52-day-old *DMD*-modified pig and wild-type pig were lysed in lysis buffer (10% 2-mercaptoethanol, 10% glycerol, 0.5 M Tris-HCl, and 1% sodium dodecyl sulfate). Dystrophin and GAPDH were separated on 4% and 8% SDS-polyacrylamide gel, respectively. Subsequently, the protein was transferred to polyvinylidene fluoride membranes. The membranes were blocked and incubated with anti-Dystrophin antibody and anti-GAPDH antibody overnight, followed by combination with horseradish peroxidase-labeled secondary antibody at room temperature for 2 h, followed by visualization of dystrophin and GAPDH using the ECL detection system (GE Healthcare Biosciences; Piscataway, NJ, USA).

### 4.8. Hematoxylin and Eosin (H&E) Staining

The tissues from founder A and wild-type pigs were fixed in Bouin’s solution and 4% PFA, respectively, embedded in paraffin, sectioned at 5 µm, deparaffinized, and stained with H&E. After dehydration through a graded ethanol series and clearing in xylene, the samples were mounted with neutral balsam and viewed with a bright-field by Olympus DP72 imaging system. Five fields were selected randomly in the H&E-stained smooth muscle sections for quantitative analyses. Photographs were taken under a microscope equipped with a digital camera (DP72; Olympus). The thickness of the smooth muscle in the stomach and intestine, per section, was calculated using IPP software (ver. 6.0; Media Cybernetics, Silver Spring, MD, USA).

### 4.9. Morphometric Analysis of Myofibers

Morphometric analysis were conducted on H&E stained cross-section of biceps femoris muscle of a 52-day-old *DMD*-modified pig, and aged-matched wild-type pig. For quantification of muscle fiber sizes, at least 5 locations were taken in the sections. The minimal feret’s diameters of all muscle fiber, excluding the fiber with central nuclei at cross-section were calculated using IPP software (ver. 6.0; Media Cybernetics, Silver Spring, MD, USA).

## 5. Conclusion

Taken together, we reported the successful targeting of *DMD* gene using CRISPR/Cas9 system in miniature pig. Our results suggested that the *DMD*-modified pig, used as dystrophy disease model, will have significant impact on the understanding of pathology and therapeutic testing.

## Supplementary Materials

Supplementary materials can be found at www.mdpi.com/1422-0067/17/10/1668/s1.

## References

[1] Koenig M., Monaco A.P., Kunkel L.M. (1988). The complete sequence of dystrophin predicts a rod-shaped cytoskeletal protein. Cell.

[2] Hoffman E.P., Brown R.H., Kunkel L.M. (1987). Dystrophin: The protein product of the Duchenne muscular dystrophy locus. Cell.

[3] Kinali M., Arechavala-Gomeza V., Cirak S., Glover A., Guglieri M., Feng L., Hollingsworth K.G., Hunt D., Jungbluth H., Roper H. (2011). Muscle histology vs. MRI in Duchenne muscular dystrophy. Neurology.

[4] Axelsson E., Ratnakumar A., Arendt M.L., Maqbool K., Webster M.T., Perloski M., Liberg O., Arnemo J.M., Hedhammar A., Lindblad-Toh K. (2013). The genomic signature of dog domestication reveals adaptation to a starch-rich diet. Nature.

[5] Mendell J.R., Shilling C., Leslie N.D., Flanigan K.M., al-Dahhak R., Gastier-Foster J., Kneile K., Dunn D.M., Duval B., Aoyagi A. (2012). Evidence-based path to newborn screening for Duchenne muscular dystrophy. Ann. Neurol..

[6] Hilton D. (2007). Muscle biopsy: A practical approach. Neuropath. Appl. Neurobiol..

[7] Vainzof M., Ayub-Guerrieri D., Onofre P.C., Martins P.C., Lopes V.F., Zilberztajn D., Maia L.S., Sell K., Yamamoto L.U. (2008). Animal models for genetic neuromuscular diseases. J. Mol. Neurosci..

[8] Beytia L., Vry J., Kirschner J. (2012). Drug treatment of Duchenne muscular dystrophy: Available evidence and perspectives. Acta Myol..

[9] Nowak K.J., Davies K.E. (2004). Duchenne muscular dystrophy and dystrophin: Pathogenesis and opportunities for treatment. EMBO Rep..

[10] Banks G.B., Chamberlain J.S. (2008). The value of mammalian models for duchenne muscular dystrophy in developing therapeutic strategies. Curr. Top. Dev. Biol..

[11] Nakamura A., Takeda S. (2011). Mammalian models of Duchenne Muscular Dystrophy: Pathological characteristics and therapeutic applications. J. Biomed. Biotechnol..

[12] Nakamura K., Fujii W., Tsuboi M., Tanihata J., Teramoto N., Takeuchi S., Naito K., Yamanouchi K., Nishihara M. (2014). Generation of muscular dystrophy model rats with a CRISPR/Cas system. Sci. Rep..

[13] Jacob H.J. (1999). Functional genomics and rat models. Genome Res..

[14] Fahrenkrug S.C., Blake A., Carlson D.F., Doran T., Eenennaam A., Faber D., Galli C., Gao Q., Hackett P.B., Li N. (2010). Precision genetics for complex objectives in animal agriculture. J. Anim. Sci..

[15] Tan W.S., Carlson D.F., Walton M.W., Fahrenkrug S.C., Hackett P.B. (2012). Precision editing of large animal genomes. Adv. Genet..

[16] Niu Y., Shen B., Cui Y., Chen Y., Wang J., Wang L., Kang Y., Zhao X., Si W., Li W. (2014). Generation of gene-modified cynomolgus monkey via Cas9/RNA-mediated gene targeting in one-cell embryos. Cell.

[17] Wang Y., Du Y., Shen B., Zhou X., Li J., Liu Y., Wang J., Zhou J., Hu B., Kang N. (2015). Efficient generation of gene-modified pigs via injection of zygote with Cas9/sgRNA. Sci. Rep..

[18] Wang X., Yu H., Lei A., Zhou J., Zeng W., Zhu H., Dong Z., Niu Y., Shi B., Cai B. (2015). Generation of gene-modified goats targeting MSTN and FGF5 via zygote injection of CRISPR/Cas9 system. Sci. Rep..

[19] Oishi I., Yoshii K., Miyahara D., Kagami H., Tagami T. (2016). Targeted mutagenesis in chicken using CRISPR/Cas9 system. Sci. Rep..

[20] Zou Q., Wang X., Liu Y., Ouyang Z., Long H., Wei S., Xin J., Zhao B., Lai S., Shen J. (2015). Generation of gene-target dogs using CRISPR/Cas9 system. J. Mol. Cell. Biol..

[21] Peng J., Wang Y., Jiang J., Zhou X., Song L., Wang L., Ding C., Qin J., Liu L., Wang W. (2015). Production of human albumin in pigs through CRISPR/Cas9-mediated knockin of human cDNA into swine albumin locus in the zygotes. Sci. Rep..

[22] Hu W., Lian L., Su B., Zhang Y. (1998). Genetic diversity of Yunnan local pig breeds inferred from blood protein electrophoresis. Biochem. Genet..

[23] Sun H., Guo T., Liu L., Yu Z., Xu W., Chen W., Shen L., Wang J., Dou X. (2010). Ischemic postconditioning inhibits apoptosis after acute myocardial infarction in pigs. Heart Surg. Forum.

[24] Mali P., Esvelt K.M. (2013). Cas9 as a versatile tool for engineering biology. Nat. Methods.

[25] Shen B., Zhang J., Wu H., Wang J., Ma K., Li Z., Zhang X., Zhang P., Huang X. (2013). Generation of gene-modified mice via Cas9/RNA-mediated gene targeting. Cell Res..

[26] Emery A.E. (2002). The muscular dystrophies. Lancet.

[27] Rona R.J., Beech R., Mandalia S., Donnai D., Kingston H., Harris R., Wilson O., Axtell C., Swan A.V., Kavanagh F. (1994). The influence of genetic counselling in the era of DNA testing on knowledge, reproductive intentions and psychological wellbeing. Clin. Genet..

[28] Shen B., Zhang W., Zhang J., Zhou J., Wang J., Chen L., Wang L., Hodgkins A., Iyer V., Huang X. (2014). Efficient genome modification by CRISPR-Cas9 nickase with minimal off-target effects. Nat. Methods.

[29] Kornegay J.N., Childers M.K., Bogan D.J., Bogan J.R., Nghiem P., Wang J., Fan Z., Howard J.F., Schatzberg S.J., Dow J.L. (2012). The paradox of muscle hypertrophy in muscular dystrophy. Phys. Med. Rehabil. Clin. N. Am..

[30] Sharp N.J., Kornegay J.N., van Camp S.D., Herbstreith M.H., Secore S.L., Kettle S., Hung W.Y., Constantinou C.D., Dykstra M.J., Roses A.D. (1992). An error in dystrophin mRNA processing in golden retriever muscular dystrophy, an animal homologue of Duchenne muscular dystrophy. Genomics.

[31] Carpenter J.L., Hoffman E.P., Romanul F.C., Kunkel L.M., Rosales R.K., Ma N.S., Dasbach J.J., Rae J.F., Moore F.M., McAfee M.B. (1989). Feline muscular dystrophy with dystrophin deficiency. Am. J. Pathol..

[32] Gaschen F.P., Hoffman E.P., Gorospe J.R., Uhl E.W., Senior D.F., Cardinet G.H., Pearce L.K. (1992). Dystrophin deficiency causes lethal muscle hypertrophy in cats. J. Neurol. Sci..

[33] Cros D., Harnden P., Pellissier J.F., and Serratrice G. (1989). Muscle hypertrophy in Duchenne muscular dystrophy. A pathological and morphometric study. J. Neurol..

[34] Wei H., Qing Y., Pan W., Zhao H., Li H., Cheng W., Zhao L., Xu C., Li H., Li S. (2013). Comparison of the efficiency of Banna miniature inbred pig somatic cell nuclear transfer among different donor cells. PLoS ONE.

[35] Wang Y., Zhou X.Y., Xiang P.Y., Wang L.L., Tang H., Xie F., Li L., Wei H. (2014). The meganuclease I-SceI containing nuclear localization signal (NLS-I-SceI) efficiently mediated mammalian germline transgenesis via embryo cytoplasmic microinjection. PLoS ONE.

[36] Whitelaw C.B., Radcliffe P.A., Ritchie W.A., Carlisle A., Ellard F.M., Pena R.N., Rowe J., Clark A.J., King T.J., Mitrophanous K.A. (2004). Efficient generation of transgenic pigs using equine infectious anaemia virus (EIAV) derived vector. FEBS Lett..

[37] Jiang H., Wong W.H. (2008). SeqMap: Mapping massive amount of oligonucleotides to the genome. Bioinformatics.

