# Supplementary material for: Porcine Zygote Injection with Cas9/sgRNA Results in DMD-Modified Pig with Muscle Dystrophy
Source: Int J Mol Sci. 2016 Oct 9;17(10):1668. doi: 10.3390/ijms17101668 (PMC5085701; doi:10.3390/ijms17101668)
Supplement: Supplementary file 1 [file ijms-17-01668-s001.pdf]

Supplementary Materials: Porcine Zygote Injection with Cas9/sgRNA Results in *DMD*-Modified Pig with Muscle Dystrophy

Hong-Hao Yu, Heng Zhao, Yu-Bo Qing, Wei-Rong Pan, Bao-Yu Jia, Hong-Ye Zhao, Xing-Xu Huang and Hong-Jiang Wei

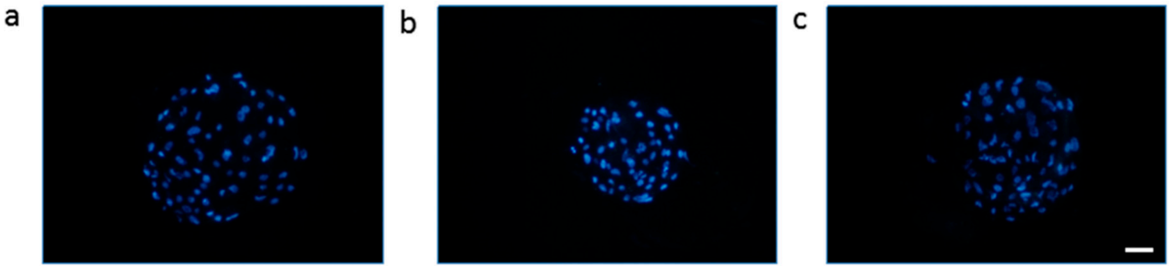

Figure S1. Nuclear staining of parthenogenetic blastocysts using Hochest33342. (**a**) Representative nuclear staining of Cas9/sgRNA-injected parthenogenetic blastocysts; (**b**) Representative nuclear staining of H_2O-injected parthenogenetic blastocysts; (**c**) Representative nuclear staining of untreated parthenogenetic blastocysts. Scale bar: 100 μm.

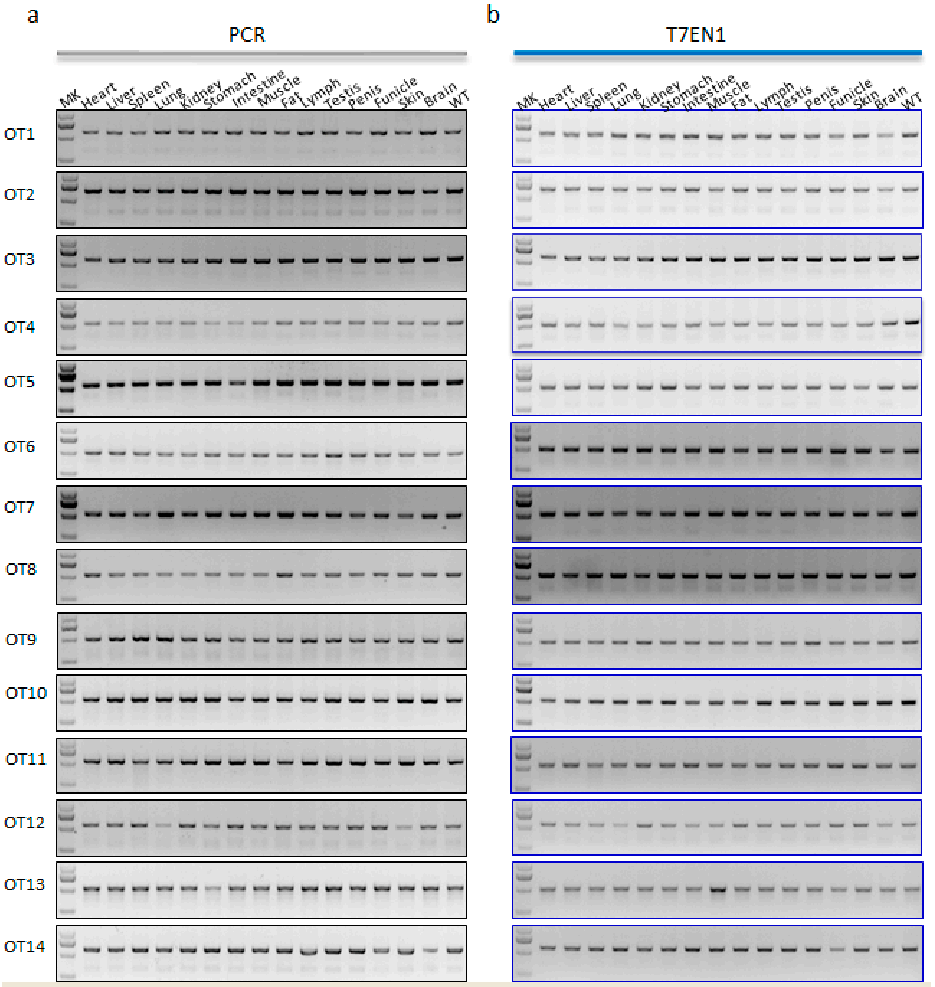

Figure S2. Detection of the *DMD* sgRNA:Cas9-mediated off-target in *DMD*-modified pig. (**a**) PCR products of the off-targeted region of *DMD* amplified from different tissues; (**b**) Detection of sgRNA:Cas9-mediated off-target cleavage of *DMD* from different tissues by T7EN1 cleavage assay.

Table S1. Summary of parthenogenetic embryos microinjected with Cas9 mRNA and sgRNA.

Group	Number of Collected Ovaries	Number of Cultured Cocs	Number of Mature Oocytes	Number of Activation Embryos	Cleavage Rate (%)	Blastocyst Development Rate (%)	Cell Number of Blastocyst Embryo
Cas9/sgRNA injection	152	759	455	400	75 (300/400)	20 (60/300)	55.5
H_2O injection	113	451	262	240	68 (163/240)	17.8 (29/163)	58.7
Untreated	66	365	230	200	78 (156/200)	35.2 (55/156)	57.5

COC, cumulus-oocyte complex.

Table S2. Summary of zygotes microinjected with the CRISPR/Cas9 system and embryo transfer.

Zygote Donor Number	Collected Zygotes Number	Recipient Number of Embryo Transfer	Number of Deliveries	Number of Offspring	*DMD* Modification Number
19	98	8	1	2	1

Table S3. Summary of the *DMD*-sgRNA:Cas9-mediated on-target efficiency (%) in different tissues of founder A.

Mutation Type	Heart	Liver	Spleen	Lung	Kidney	Stomach	Intestine	Muscle	Fat	Lymph	Testis	Penis	Funicle	Skin	Brain
Mutant 1 (−11)	10	20	10	20	10	0	0	40	10	30	0	0	20	10	30
Mutant 2 (−36)	0	30	20	0	0	40	20	10	10	0	20	30	0	50	20
Mutant 3 (−5,+14)	40	10	10	10	30	20	20	0	0	0	30	30	10	0	30
Mutant 4 (−6, +16)	0	0	0	0	0	0	0	20	0	0	0	0	0	0	0
Total	50	60	40	30	40	60	40	70	20	30	50	60	30	60	80

Table S4. List of putative off-target sites homologous to sgRNA.

	20	19	18	17	16	15	14	13	12	11	10	9	8	7	6	5	4	3	2	1	N	G	G	Chro.	Location	Strand
DMD sg1	G	T	T	G	G	A	G	A	C	T	G	A	A	G	T	A	A	A	C	C	T	G	G	X	31396957	+
OT1	G	T	G	G	G	A	G	A	G	G	G	A	A	G	T	A	A	A	C	C	T	G	G	3	98663037	−
OT2	G	T	G	G	G	A	G	A	G	G	G	A	A	G	T	A	A	A	C	C	T	G	G	3	98588326	−
OT3	G	G	A	G	G	A	A	A	C	G	G	A	A	G	T	A	A	A	C	C	T	G	G	12	36178851	+
OT4	C	T	T	G	G	T	G	C	C	T	C	A	A	G	T	A	A	A	C	C	A	G	G	6	90817452	+
OT5	G	T	T	G	G	G	G	A	A	T	G	T	A	G	T	A	A	A	C	C	A	G	G	1	168736398	+
OT6	C	T	T	G	G	G	G	G	C	T	G	C	A	G	T	A	A	A	C	C	A	G	G	12	36176812	−
OT7	G	C	T	G	G	A	G	A	C	T	G	A	A	G	A	A	A	A	C	A	T	G	G	7	33407843	+
OT8	A	T	T	G	T	A	A	A	C	T	G	A	T	G	T	A	A	A	C	C	A	G	G	16	32234398	−
OT9	T	T	T	T	G	A	G	A	A	T	G	A	A	G	T	A	A	A	T	C	A	G	G	8	101105802	−
OT10	T	T	T	G	G	A	G	G	C	T	G	C	A	G	T	A	G	A	C	C	A	G	G	X	3173939	−
OT11	G	T	T	G	C	T	G	A	C	T	G	A	A	G	T	T	A	A	C	C	C	G	G	1	241128345	−
OT12	G	T	C	A	G	A	G	A	C	A	G	A	A	G	T	A	A	T	C	C	T	G	G	9	9959203	+
OT13	G	T	C	A	G	A	G	A	C	A	G	A	A	G	T	A	A	T	C	C	T	G	G	9	9782547	+
OT14	T	T	T	T	G	A	G	G	C	T	G	A	A	G	T	G	A	A	C	C	G	G	G	6	47144948	+

PAM is shaded in green; Bases different to sgRNA are shaded in red.

Table S5. Oligonucleotides for generating sgRNA expression vector.

Oligonucleotides	Sequence
DMD-sgRNA top strand	5′-TAGGGTTGGAGACTGAAGTAAACC-3′
DMD-sgRNA bottom strand	5′-AAACGGTTTACTTCAGTCTCCAAC-3′

Table S6. Primers for genotyping and amplifying the Cas9/sgRNA targeted fragment.

Name	Sequence	Amplicon
pDMD For	5′-GAAGGCTTATTATTGTATGTG-3′	531
pDMD Rev	5′-TCAAGAGTTATTCTCCAAAGG-3′	

Table S7. Sequences of primers for PCR amplification of the off-target sites.

Name	Sequence	Amplicon
OT1 For	5′-TAGGTAAAGGTGAGGCACAGTAAAC-3′	578
OT1 Rev	5′-AACAGCACTAACAGAACTGATCGAC-3′	
OT2 For	5′-AACGAATTCGACTAGGAACTCTGAG-3′	636
OT2 Rev	5′-TATTCAAAGATGTGTGAGGAGGAGC-3′	
OT3 For	5′-TTCTTTCTGCGTTCTATGCTTGCTG-3′	556
OT3 Rev	5′-CTTCTGGACAGTTTATTTGAGGAGG-3′	
OT4 For	5′-ACACATCTCCAAGTTTCGGTCTCTG-3′	515
OT4 Rev	5′-CTGGTTATGAGTCTCTGTCTCTTAG-3′	
OT5 For	5′-TCCTTTGTGCTATATGCTAGACTCC-3′	525
OT5 Rev	5′-ATACAAATCCCCAAAGAAGCAACAG-3′	
OT6 For	5′-GGCTCTGGAGAAAGCTACTGCAC-3′	475
OT6 Rev	5′-TTGGATCTGGCTGTTTCCTGGTC-3′	
OT7 For	5′-CTGTGACAGATGGACTCTAGAAATG-3′	536
OT7 Rev	5′-ATAGTGGGTTAAGGATCTGGCATTG-3′	
OT8 For	5′-TGAGAATCATAAAACATCAGGTCGC-3′	510
OT8 Rev	5′-CTCTCTTCTGAACTGATTCTAGGTG-3′	
OT9 For	5′-AATACAGGTGACAACAACTCAAGAC-3′	496
OT9 Rev	5′-TAATTGGCTTATACATTCATGGGTG-3′	
OT10 For	5′-CCTTGAGATTTCCAGCATAAGACAC-3′	511
OT10 Rev	5′-ACCCCGCTTCTTCATTAAGTTCTAG-3′	
OT11 For	5′-ACAGATAGGGTCATCCATTTTCAAG-3′	534
OT11 Rev	5′-TACATTCAGGATCTCTGCTTTGCTC-3′	
OT12 For	5′-AAAAAGCAAGTGTCCGCAGATTCAG-3′	541
OT12 Rev	5′-TTATGAGATTCACATGAAGGCAGCC-3′	
OT13 For	5′-TCTGTGCTCGCAACTTGGTTCTCTC-3′	508
OT13 Rev	5′-CTTTCAACGACGACAAGGCAGATTC-3′	
OT14 For	5′-CAGTTTCCTCAGGTCTCAGCTATTG-3′	584
OT14 Rev	5′-TCTGTCCTCTCGCTTTACTTGTGTC-3′	
